# Systematic Evaluation of DNA Sequence Variations on *in vivo* Transcription Factor Binding Affinity

**DOI:** 10.3389/fgene.2021.667866

**Published:** 2021-09-09

**Authors:** Yutong Jin, Jiahui Jiang, Ruixuan Wang, Zhaohui S. Qin

**Affiliations:** ^1^Department of Biostatistics and Bioinformatics, Emory University, Atlanta, GA, United States; ^2^College of Environmental Sciences and Engineering, Peking University, Beijing, China

**Keywords:** non-coding variant annotation, transcription regulation, transcription factor binding motif, gapped k-mer SVM classifier, genome-wide association study, position weight matrix

## Abstract

The majority of the single nucleotide variants (SNVs) identified by genome-wide association studies (GWAS) fall outside of the protein-coding regions. Elucidating the functional implications of these variants has been a major challenge. A possible mechanism for functional non-coding variants is that they disrupted the canonical transcription factor (TF) binding sites that affect the *in vivo* binding of the TF. However, their impact varies since many positions within a TF binding motif are not well conserved. Therefore, simply annotating all variants located in putative TF binding sites may overestimate the functional impact of these SNVs. We conducted a comprehensive survey to study the effect of SNVs on the TF binding affinity. A sequence-based machine learning method was used to estimate the change in binding affinity for each SNV located inside a putative motif site. From the results obtained on 18 TF binding motifs, we found that there is a substantial variation in terms of a SNV’s impact on TF binding affinity. We found that only about 20% of SNVs located inside putative TF binding sites would likely to have significant impact on the TF-DNA binding.

## Introduction

Thousands of genome-wide association studies (GWAS) have been conducted over the past 15years, resulting in considerable single nucleotide variants (SNVs) being discovered as robustly associated with a wide array of phenotypes (Welter et al., 2014). The vast majority of the trait-associated variants detected by these studies lies in the non-coding part of the human genome (Maurano et al., 2012) and is hypothesized to play a regulatory role in controlling the expression of genes related to disease pathogenesis. Moreover, it has been demonstrated that GWAS-identified variants are enriched in regulatory regions (Cookson et al., 2009).

A possible mechanism for non-coding trait-associated variants is that mutations may affect the *in vivo* binding of transcription factors (TFs) to regulatory elements (promoters and enhancers; Pasquali et al., 2014) by disrupting the canonical motif pattern recognized by the TF (Yan et al., 2021). Here, identifying TF binding sites is a well-known classical bioinformatics problem. Many statistical and computational approaches have been proposed (Lawrence et al., 1993; Hertz and Stormo, 1999). Despite the fact that many sophisticated methods have been proposed lately to model TF binding motif accurately (Zhao et al., 2012; Mathelier and Wasserman, 2013; Eggeling et al., 2015; Keilwagen and Grau, 2015; Siebert and Söding, 2016), in practice, using position weight matrix (PWM; Stormo et al., 1982) scan is still the most commonly used method for identifying TF binding sites, due to its simplicity. PWM models the nucleotide preference at each position independently. There are different forms of PWMs, like nucleotide frequencies, probability, or log likelihood. In this study, either frequency or probability form of the PWM was imported from the original databases, but we transformed all the frequency form of PWM to the probability form ahead of all analyses. By scanning the genome, one can calculate a matching score based on the PWM for each candidate binding site. A locus with score exceeding a pre-defined threshold is considered to be a putative binding site. Subsequently, all mutations found within such binding sites are considered as consequential and marked (Boyle et al., 2012; Ward and Kellis, 2012). However, it is well-known that many positions within a TF binding motif are weakly conserved and the functional impact of a mutation at such positions are likely to be low. Although there exist highly informative PWMs for many TF binding sites in databases like TRANSFAC (Wingender et al., 2000), Factorbook (Wang et al., 2012), JASPAR (Sandelin et al., 2004), HOCOMOCO (Kulakovskiy et al., 2017), and CIS-BP, these resources are designed for characterizing motif patterns. Their effectiveness for measuring the impact of mutations has yet to be investigated. In this study, we aim to evaluate whether measuring the overall PWM probability difference for a motif with or without a mutation is a reasonable strategy to measure the impact of the mutation.

In a recent study, Ghandi et al. (2014) developed a novel sequence-based computational method to predict the impact of regulatory variants. The authors took advantage of sequencing-based assays, such as ChIP-seq (Johnson et al., 2007) that is able to recognize TF binding *in vivo*, to define gapped k-mer support vector machine (gkm-SVM) weights to quantify the different level of abundance of k-mers at functionally important genomic loci. The authors then defined deltaSVM scores as the induced change in the SVM weights and used deltaSVM score to quantify the functional impact of SNVs. Applications of deltaSVM showed accurate prediction of the impact of SNVs on DNase I sensitivity in the genomic context (Lee et al., 2015).

Built on its success on regulatory sequence prediction, a natural extension is to apply the gkm-SVM methodology to predict TF binding. Given the dominance of PWM in this area, it is of great interest to compare PWM scores with SVM weights on the same DNA fragment. In this work, we conducted a survey to compare these two motif-scoring methods. Subsequently, we compared the consistency of using PWM to evaluate the impact of SNVs on TF binding *in vivo* with that from deltaSVM. We first employed gkm-SVM (Ghandi et al., 2014) to evaluate the TF binding potential for all 10-mers based on the TF’s ChIP-seq data. We then quantify the effect of a SNV on the TF binding using deltaSVM. We believe that deltaSVM scores derived from ChIP-seq data can serve as a useful resource to quantify the impact of SNVs throughout the genome. Finally, we compare the SNV impact measured by deltaSVM with that of the probability difference derived from the PWM. The goal is to check whether the results derived from these two methods are comparable.

## Materials and Methods

### Data Sources

In this study, we surveyed 18 TFs including BCL11A, CTCF, EGR1, GABPA, JUN, JUND, MAX, NANOG, POU5F1, RAD21, RFX5, SIX5, SRF, STAT1, TCF12, USF1, USF2, and YY1. We choose these 18 TFs since their motif PWMs are well-defined and their ChIP-seq data are available from the Encyclopedia of DNA Elements (ENCODE) consortium (ENCODE Project Consortium, 2012). The PWMs used in this study are obtained from JASPAR and Factorbook. The IDs of these PWMs are summarized in Supplementary Table S1.

All the ChIP-seq peak region information is provided by ENCODE and downloaded from the ENCODE website. The dataset IDs are summarized in Supplementary Table S2.

### Measuring TF Binding Strength

Using classical approaches, the binding affinity of the TF is measured by the probability calculated based on the PWM of the TF. And, the impact of a SNV can be evaluated by the difference between the probabilities of the two motif incidences (differing at the SNV position). An alternative method is based on the new gkm-SVM method introduced recently (Ghandi et al., 2014). The deltaSVM scores derived from there (Lee et al., 2015) can be used to measure the impact of a SNV.

Motif incidences are typically identified by sliding through the entire human genome using a pre-defined motif PWM to calculate a matching probability for each possible motif start position. Mathematically, the PWM model assumes a product-multinomial model. Strictly speaking, a PWM model is better defined as an inhomogeneous Markov model of order zero. Nevertheless, a motif PWM score is defined as the negative log-transformed probability that the DNA motif is generated from the series of underlying multinomial distributions defined by the PWM. Here, we use CTCF (motif length 15bp) as an example: adopting the same PWM and the same threshold for calling a match described previously (Xu et al., 2015), we identified 139,084 15-mer CTCF motif sites genome-wide. Among them, there are 48,804 unique 15-mer motif sequences. For a 15-bp motif like CTCF motif, since it contains six different alignments (overlapping bases 1–10, 2–11, 3–12, 4–13, 5–14, and 6–15) for a 10-mer, we assessed the probability of observing the 10-mer using part of the PWM for each of the six potential matching alignments (positions 1–10, 2–11, 3–12, 4–13, 5–14, and 6–15), and then selected the highest probability among the six as the probabilistic value of the specific 10-mer, defined as the 10-mer PWM score. We use the aforementioned strategy described above to process motifs longer than 10bp. No such alignment is needed for motif with 10bp in length.

In some scenarios, relative entropy (Vinga, 2014) is preferred over the PWM score as the estimate of the binding strength. The relative entropy for motif sequence (*a*_1_, *a*_2_,…,*a_L_*) is defined by

$$-\overset{L}{\underset{i=1}{\sum }}p_{i,a_{i}}.\;\;\log\left(p_{i,a_{i}}/p_{a_{i}}\right),$$

where *a_i_*=*A*, *C*, *G* or *T*, *L* is the motif length. *P_i,j_* is the probability for nucleotide *j* at position *i*. *P_j_*’s are background probabilities, which are fixed at 0.25 in the present study as in most applications, *J*=*A*, *C*, *G* or *T*. Higher values of the entropy indicate better fit to the PWM model.

Alternatively, gkm-SVM (Ghandi et al., 2014) can be used to measure how likely a DNA segment may be bound by a TF. Using ChIP-seq data, we first treated peak regions annotated by ENCODE as the positive training set, whereas regions outside peaks are selected as the negative training set. These null sequences were generated using the genNullSeqs function, part of the gkmSVM R package (Ghandi et al., 2014). Here, the tolerance parameters for difference in repeat ratio, GC content and length were all set to 0.02, such that null sequences generated resembles the input positive regions. Next, we applied gkm-SVM to estimate the SVM weights of all possible 10-mers. Because TF binding is cell type-specific, the 10-mer SVM weights are different from one cell type to another. However, we found that there are about 80% overlaps among the top 1,000 10-mers with the highest SVM weights from the three cell types (GM12878, K562, and H1). And, the percentage increases to 84% for the top 500 10-mers, so we concluded that the SVM weights for CTCF obtained from the three different cell types are quite consistent. Therefore, for all subsequent analyses, we used SVM weights calculated from ChIP-seq data collected from the GM12878 cell line. Note that, although there is little difference for the SVM weight in these three cell lines, it does not mean this is the case for other cell lines / tissues types and for other TFs.

With the defined PWM scores and SVM weights, we can compare sensitivity and specificity of *in vivo* TF binding predicted by these two quantities by adopting various thresholds, and then can enumerate the number of false positives and false negatives.

### Measuring Impact of SNV on TF Binding Strength

If a motif site contains a SNV, we define the motif with the reference allele at the SNV position to be the wild-type (WT) motif and the motif with the alternative allele at the SNV position to be the variant motif. The difference of motif PWM scores between the WT motif and the variant motif is defined as the delta-PWM score of the motif.

**Figure 1:**
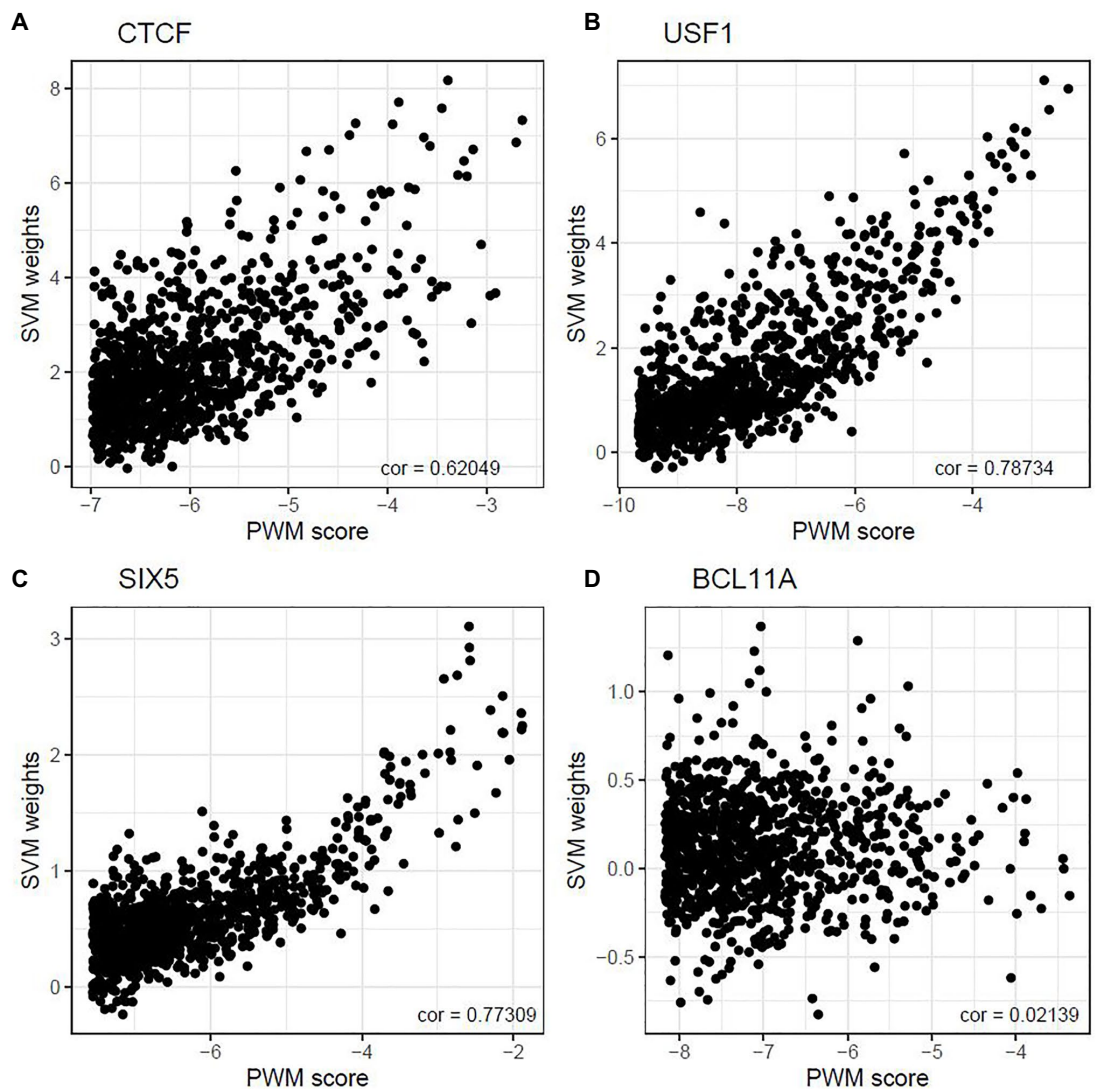
Correlation between top-ranked 10-mer’s PWM score and their weight. The correlations between 10-mer PWM scores and SVM weights were 0.620, 0.787, 0.773, and 0.021 for CTCF **(A)**, USF1 **(B)**, SIX5 **(C)**, and BCL11A **(D)** in GM12878, respectively.

Alternatively, for each SNV, sliding along its flanking sequence, there are 10 different 10-mers containing this SNV. Assuming the SNV is bi-allelic, as in the original study, the deltaSVM score of the SNV is defined as the sum of the SVM weight differences between 10 pairs of corresponding WT and variant motifs, as illustrated in Figure 1 in the original study (Lee et al., 2015).

For each motif, in order to determine an empirical threshold for calling an SNV impactful on TF binding, we first randomly selected 10,000 non-motif sites of the same length (below PWM scan probability threshold) as the control set. For each base, we calculated the average deltaSVM score over three possible variants. After repeating this step for all positions in the control set, we established a large collection of averaged deltaSVM scores from random sequences. We then determined the 2.5 percentile and the 97.5 percentile of the empirical distribution of the deltaSVM scores as the significant thresholds.

## Results

It is important to annotate non-coding variants. Currently, all SNVs that fall into putative TF binding sites, called by PWM scan, are considered to affect transcriptional regulation. In this study, using the newly developed gkm-SVM method and ChIP-seq data, we conducted a survey to tell whether these SNVs indeed affect TF binding *in vivo*. In the present study, we utilized data from the ENCODE project (ENCODE Project Consortium, 2012). For each TF, using peaks called from its ChIP-seq data on the GM12878 cell line, we first divided the genome into two categories: peaks and non-peaks. Next, we counted the occurrences of every 10-mer found in the ENCODE peak regions and obtained its SVM weights. Then for the top 10mers based on PWM scores, we conducted a comprehensive survey of the impact of all common SNVs occurring within each of these 10mers. By common SNVs, we mean all SNVs listed in the dbSNP153 Common SNPs panel (accessed from the UCSC TableBrowser). This strategy was applied to all 18 TFs.

### Correlation Between SVM Weights and PWM Scores

It is of interest to find out which type of scores is better to detect TF binding *in vivo*. Therefore, we conducted the following comparison study on CTCF and JUND. We used ENCODE ChIP-seq data obtained from the K562 cell line, which differs from the GM12878 cell line used to train the SVM weights. We first obtained 1,000 top ranked sequences under ChIP-seq peaks, which are 244bps in length for CTCF and 280bps in length for JUND. Then for each TF, we randomly extracted 1,000 sequences of the same length outside peak regions as controls. Within these sequences, full length motif PWM scores and 10-mer SVM weights were retrieved for all possible motif incidences. We used two difference methods to summarize all the scores in each sequence: average and maximum. The performance of the two approaches was evaluated using area under the curve (AUC) of receiver operator characteristic (ROC) and precision and recall curve (PRC) and is summarized in Supplementary Table S3. From the results, we observed that PWM score performed better for CTCF when using the maximum scores, and SVM weight performed better in all other cases. The results suggest that SVM weight is a competitive method to score TF binding *in vivo* and performs better than PWM scores when the motif is shorter and weak.

Next, we address the question about the relationship between the PWM scores and the SVM weights. For each TF, we first calculated the PWM scores for all 10-mers and selected 1,000 10-mers with the highest PWM scores. Then we calculated the SVM weights for each of the 1,000 10-mers. We found that the correlation between PWM scores and SVM weights of these 10-mers ranges from −0.081 to 0.787. Figure 1 shows the scatter plots for four TF binding motifs: CTCF, USF1, SIX5, and BCL11A. The complete set of results for all 18 TFs is summarized in Supplementary Figure 1. For some TFs, such as USF1 and SIX5, moderately strong and positive correlation relationships are observed between the two measures, whereas such a trend is less obvious in other TFs, such as BCL11A and STAT1. Other TFs, such as CTCF, lie in between. This suggests the PWM-based method and SVM-based method does not always agree when measuring *in vivo* TF binding strength. The complete summary of the top 100 motif incidences, in terms of deltaSVM scores for all 18 TFs are summarized in Supplementary Table S4. To further illustrate this point, we selected the top 20 10-mers according to their SVM weights and displayed both their SVM weights and PWM scores in Supplementary Figure 2. In this part, binding strength is calculated using the relative entropy for the purpose of better visualization. As can be seen, some of the 10-mers with high SVM weights do not show a very significant PWM score, especially for JUND, MAX, POU5F1, and USF1. The observed discrepancy often shows that the core motif is often well-understood, but the motif length and the exact boundary of the motif are often debatable. On the other hand, focusing on 10-mer frequencies and weights can overcome this issue.

### Exploring Potential Association Between TFs and Complex Diseases

We next conducted a comprehensive survey of complex disease-associated SNVs in terms of their impact on TF binding. We hypothesize that if a TF is playing an important role in the pathogenesis of a disease, then SNVs that affects the binding of the TF will be enriched among those disease-associated SNVs. We used both PWM-based method and gkm-SVM-based method and compared their findings. We studied 11 diseases including Alzheimer’s disease (AD), asthma, breast cancer, cardiovascular diseases, child development disorders pervasive (CDDP), colorectal cancer, Crohn’s disease, lung cancer, obesity, psoriasis, and type 2 diabetes. For each disease, we first identified all disease-associated SNVs from the PheGenI web portal (Ramos et al., 2014) using values of *p* of 10^−6^ as the threshold. Since PheGenI only collect index SNVs, which means the actual functional SNV may be a nearby SNV that is in high linkage disequilibrium (LD) with it. Hence, we included all SNVs located within 5kb of the GWAS index SNV, hoping to capture the functional SNV(s).

**Figure 2:**
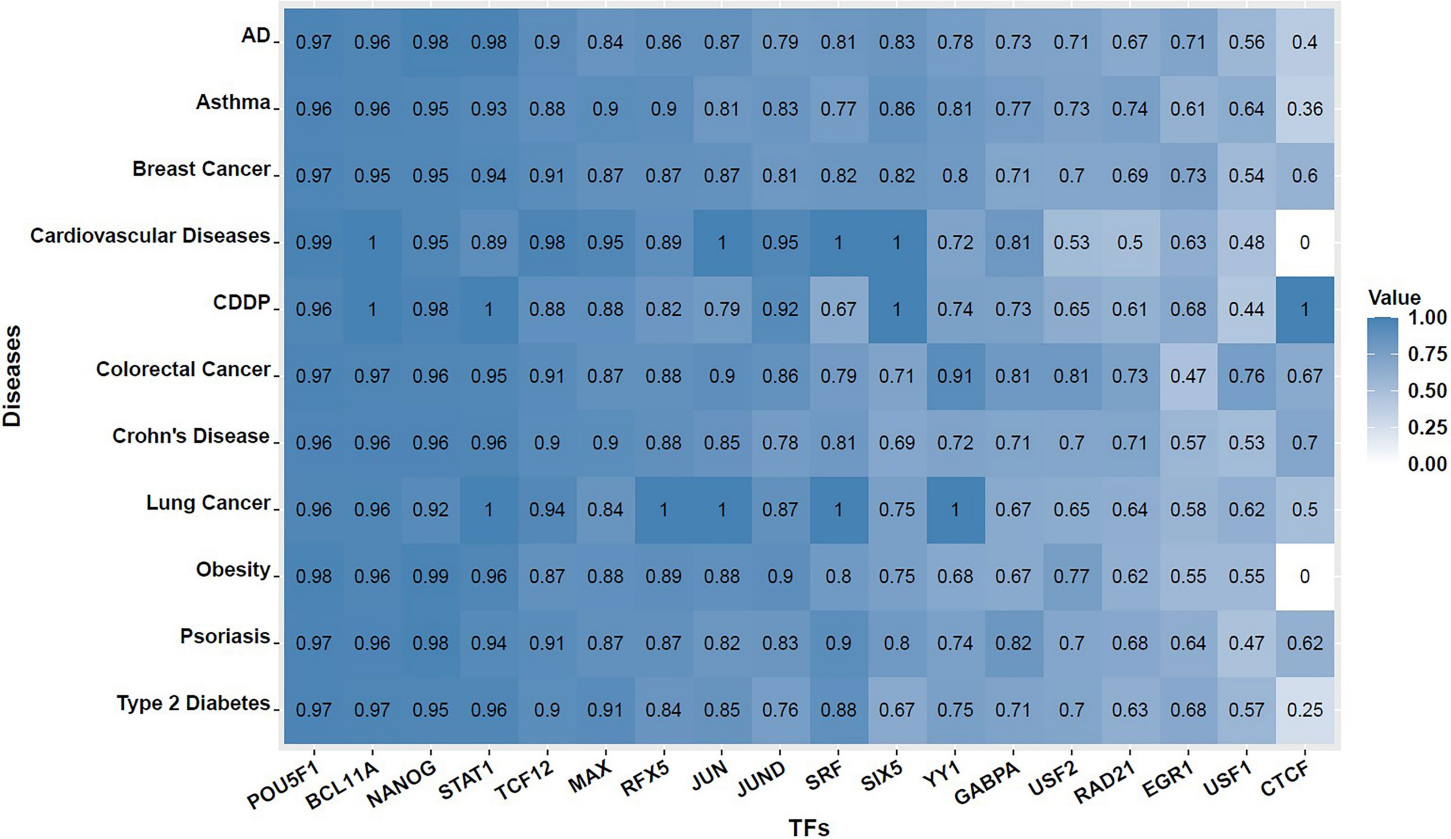
Heat maps showing percentage of discordant SNVs across 18 TFs and 11 complex diseases. That is, among all SNVs that overlap with putative TF binding sites identified by PWM at GWAS-identified disease loci, what proportion of them has insignificant deltaSVM scores. The value shown inside each cell is the percentage of discordant SNVs found for the TF and disease combination.

Next, for each TF, we went through every one of the SNVs at the disease-associated loci to see whether it overlaps with any putative TF binding site according to its motif’s PWM. Then, we assessed that among these SNVs, how many of them cause significant changes, i.e., with significant deltaSVM scores exceeding the empirical thresholds. SNVs that overlap with putative TF binding sites identified by PWM but have insignificant deltaSVM scores (not exceeding the significance thresholds) were named “discordant SNVs.” Here, significance is defined as exceeding the threshold corresponding to the empirical value of *p* of 0.05 (described in section Measuring Impact of SNV on TF Binding Strength of section Materials and Methods). The percentage of discordant SNVs in all 11 diseases were evaluated for each motif and presented in Supplementary Table S5, and its corresponding heatmap is shown in Figure 2. We observe that there is much variation in terms of the number of motif incidence among the 18 TFs. For some TFs, such as POU5F1, most of the SNVs overlapped with significant motif incidences identified using the traditional PWM-based methods, do not make much difference in SVM weights, which suggest that these SNVs may have limited effect on POU5F1 binding *in vivo*, and the opposite is also possible, that a SNV not identified by PWM-based methods may significantly affect POU5F1 binding *in vivo*. Despite variations among the TFs, we observe that the average percentage of discordant SNVs across 18 TFs are close to 80% for all 11 diseases we have tested. This illustrated the importance of using information beyond PWM to better study the biological impact of SNVs on complex disease etiology.

## Discussion

Understanding the functional impact of non-coding variants is a grand challenge in contemporary molecular genetics. The ever-increasing of genomics and epigenomics data provided an unprecedented opportunity to address this issue. Many attempts have been made to understand the impact of non-coding variants (Stormo et al., 1982; Kircher et al., 2014; Ritchie et al., 2014; Lee et al., 2015; Lu et al., 2015; Quang et al., 2015; Shihab et al., 2015; Zhou and Troyanskaya, 2015; Chen et al., 2016, 2019; Ionita-Laza et al., 2016; Li et al., 2016; Huang et al., 2017; Gao et al., 2018; Zhou and Zhao, 2018). In this study, we explored how to best quantify the impact of SNVs on *in vivo* TF binding strength, which may help us to better understand potential functional impact of disease-associated non-coding variants.

Existing methods to evaluate the impact of SNVs on TF binding, such as regulomeDB (Boyle et al., 2012), work by determining whether a SNV fall inside a putative TF binding sites inferred by PWM scan. Despite the fact that the PWM is a simple yet effective way to represent the canonical TF binding motif patterns, however, with few exceptions (Hu et al., 2010), most of the PWMs assume that all the positions are independent, which reflect our limited understanding of TF binding. Inspired by the gkm-SVM method (Ghandi et al., 2014; Lee et al., 2015), in this work, we suggested to consider an alternative metric based on SVM weights to measure *in vivo* TF binding affinity and to assess the functional impact of SNVs. The training data utilized is derived from ENCODE TF ChIP-seq data that measure *in vivo* TF binding. Using 18 different TFs as examples, we found that there is substantial variation in terms of a SNV’s impact on *in vivo* binding affinity. For most TFs, there is a positive, yet moderate correlation between the new SVM weight and the classical probability measure based on the PWM. However, for individual binding site, we found many with high PWM scores but low SVM weights and *vice versa*.

Additionally, we found that the vast majority of the SNVs located inside putative motifs identified by PWM scan have little effect on the TF’s *in vivo* binding affinity according to our analysis based on SVM weights. To be specific, only about 20% of SNVs located inside putative TF binding sites will likely have significant effect on the TF-DNA binding *in vivo*. This suggests that using the traditional PWM approach to annotate SNVs in terms of their impact on TF binding *in vivo* may be unreliable. Our results suggest that it is inadequate to use PWM-based probability alone to annotate SNVs for their impact on TF binding *in vivo* and suggest that more detailed elucidation of the functional impact of SNVs to be conducted.

We do not consider the SVM weight as the gold standard for measuring TF binding strength. However, we do think the deltaSVM method provide an attractive alternative to PWM-based methods that are being predominantly used in TF binding inference and also functional annotation of non-coding variants. This is because PWM model assumes different positions are independent, which is over-simplified. Given that PWM-based method dominates the practice of predicting TF binding sites, we want to caution researchers that current annotation for SNP on TF binding based on PWM may not be reliable. We recommend adding SVM-based method to PWM-based method to measure TF binding strength. We think the prediction could be much more reliable if both methods return significant results.

There are many epigenomics factors that affect TF binding *in vivo* including but not limited to DNA methylation and chromatin accessibility. We focus on studying the impact of SNVs on TF binding using only sequence information. But there are many other tools that are available to annotate the genome using such epigenomics information (Stormo et al., 1982; Kircher et al., 2014; Ritchie et al., 2014; Lee et al., 2015; Lu et al., 2015; Quang et al., 2015; Shihab et al., 2015; Zhou and Troyanskaya, 2015; Chen et al., 2016, 2019; Ionita-Laza et al., 2016; Li et al., 2016; Huang et al., 2017; Gao et al., 2018; Zhou and Zhao, 2018). Adding such information can help to provide more accurate information to study the impact of SNV on TF binding *in vivo* in specific tissues or cell types.

The predictive power of our method has limitations. The SVM weights are trained using ChIP-seq data, which are cell-type specific, and the quality of the data varies. Nevertheless, we felt that it is important that we bring in fresh new information from the latest genomics data to enrich our understanding of TF binding. Such information may shed light on the pathogenesis of GWAS findings in terms of disease mechanism and pathogenesis.

Another limitation is that currently we do not have experimental data other than ChIP-seq to compare the TF binding prediction results between these two methods. For future work, we will consider utilizing different types of epigenomics data, such as chromatin accessibility data (Pique-Regi et al., 2011) or DNA methylation data (Xu et al., 2015), to evaluate the prediction accuracy.

## Data Availability Statement

All datasets and codes for this study can be found on GitHub (https://github.com/YutongJ/TFBS).

## Author Contributions

YJ and ZSQ conceived the idea of the study. YJ, JJ, and RW performed all the analyses. YJ, JJ, and ZSQ wrote the manuscript. All authors contributed to the article and approved the submitted version.

## Funding

ZSQ was partially supported by NIH R56 AG060757. Open access publication fee was paid by Rollins School of Public Health at Emory University.

## Conflict of Interest

The authors declare that the research was conducted in the absence of any commercial or financial relationships that could be construed as a potential conflict of interest.

## Publisher’s Note

All claims expressed in this article are solely those of the authors and do not necessarily represent those of their affiliated organizations, or those of the publisher, the editors and the reviewers. Any product that may be evaluated in this article, or claim that may be made by its manufacturer, is not guaranteed or endorsed by the publisher.
