# Supplementary material for: Systematic Evaluation of DNA Sequence Variations on in vivo Transcription Factor Binding Affinity
Source: Front Genet. 2021 Sep 9;12:667866. doi: 10.3389/fgene.2021.667866 (PMC8458901; doi:10.3389/fgene.2021.667866)
Supplement: Supplementary file 1 [file Data_Sheet_1.docx]

Supplementary Material

# Supplementary Figures and Tables

## Supplementary Figures

**Supplementary Figure 1.** Pearson correlation between top-ranked 10-mer’s PWM scores and their weights for all 18 TFs. TFs are arranged by the ascending order of the Pearson correlation coefficients.

**Supplementary Figure 2.** Correlation between top-ranked 10-mer’s SVM weights and their PWM scores for all 18 TFs.

**1.2 Supplementary Tables**

| TF | Source | ID | Length of motif | Number of motif sites in hg19 |
| --- | --- | --- | --- | --- |
| BCL11A | FACTORBOOK | HGNC:13221 | 11 | 4960192 |
| CTCF | JASPAR | MA0139.1 | 15 | 139084 |
| EGR1 | JASPAR | MA0162.2 | 14 | 1264481 |
| GABPA | FACTORBOOK | HGNC:4071 | 11 | 1299241 |
| JUND | JASPAR | MA0491.1 | 11 | 4480015 |
| JUN | FACTORBOOK | HGNC:6204 | 13 | 1957333 |
| MAX | JASPAR | MA0058.2 | 10 | 5417829 |
| NANOG | FACTORBOOK | HGNC:20857 | 11 | 4960192 |
| POU5F1 | JASPAR | MA1115.1 | 11 | 13594750 |
| RAD21 | FACTORBOOK | HGNC:9811 | 15 | 589296 |
| RFX5 | JASPAR | MA0510.1 | 15 | 1165191 |
| SIX5 | FACTORBOOK | HGNC:10891 | 15 | 204422 |
| SRF | JASPAR | MA0083.2 | 18 | 519684 |
| STAT1 | JASPAR | [MA0137.3](http://jaspar.genereg.net/matrix/MA0137.3) | 11 | 4157483 |
| TCF12 | JASPAR | [MA1648.1](http://jaspar.genereg.net/matrix/MA1648.1) | 11 | 7724994 |
| USF1 | JASPAR | MA0093.2 | 11 | 2113502 |
| USF2 | JASPAR | MA0526.2 | 16 | 1244072 |
| YY1 | JASPAR | MA0095.2 | 12 | 1501423 |

**Supplementary Table S1.** The resources of 18 TFs are listed below. The last column indicates the number of candidate motif sites genome-wide. All source files can be found on GitHub.

**Supplementary Table S2.** ENCODE dataset IDs and file accessions of 18 the TFs.

| TF | File Accessions | Experiments |
| --- | --- | --- |
| BCL11A | ENCFF468MFY | https://doi.org/doi:10.17989%2FENCSR000BHA |
| CTCF | ENCFF002DAJ | https://doi.org/doi:10.17989%2FENCSR000DKV |
| CTCF  (Testing)^1^ | ENCFF085HTY | https://doi.org/doi:10.17989%2FENCSR000BPJ |
| CTCF  (Validation)^2^ | ENCFF002CDS | https://doi.org/doi:10.17989%2FENCSR000AMF |
| CTCF  (Validation)^3^ | ENCFF001XSU | https://doi.org/doi:10.17989%2FENCSR000DWE |
| EGR1 | ENCFF618EFD | https://doi.org/doi:10.17989%2FENCSR000BRG |
| GABPA | ENCFF116EXQ | https://doi.org/doi:10.17989%2FENCSR331HPA |
| JUND | ENCFF556PIY | https://doi.org/doi:10.17989%2FENCSR000DYS |
| JUND  (Testing) ^1^ | ENCFF337DKJ | https://doi.org/doi:10.17989%2FENCSR000EGN |
| JUN | ENCFF629BFI | https://doi.org/doi:10.17989%2FENCSR000EFS |
| MAX | ENCFF083KVY | https://doi.org/doi:10.17989%2FENCSR000DZF |
| NANOG | ENCFF735WFO | https://doi.org/doi:10.17989%2FENCSR061DGF |
| POU5F1 | ENCFF990CFV | https://doi.org/doi:10.17989%2FENCSR364SNE |
| RAD21 | ENCFF753RGL | https://doi.org/doi:10.17989%2FENCSR000BMY |
| RFX5 | ENCFF402FYF | https://doi.org/doi:10.17989%2FENCSR000DZW |
| SIX5 | ENCFF606WUV | https://doi.org/doi:10.17989%2FENCSR000BJE |
| SRF | ENCFF030NYG | https://doi.org/doi:10.17989%2FENCSR000BMI |
| STAT1 | ENCFF817QHW | https://doi.org/doi:10.17989%2FENCSR332EYT |
| TCF12 | ENCFF017LZE | https://doi.org/doi:10.17989%2FENCSR725VFL |
| USF1 | ENCFF805GKD | https://doi.org/doi:10.17989%2FENCSR000BGI |
| USF2 | ENCFF372DRC | https://doi.org/doi:10.17989%2FENCSR000DZU |
| YY1 | ENCFF967ACD | https://doi.org/doi:10.17989%2FENCSR000BNP |

^1^The dataset was from cell line K562 and was used to evaluate the prediction accuracy of gkm-SVM.

^2^The dataset was from cell line H1-hESC and was used to determine whether the performance of SVM weight is consistent across difference cell lines.

^3^The dataset was from cell line K562 and was used to determine whether the performance of SVM weight is consistent across difference cell lines.

**Supplementary Table S3.** The performance of PWM scores and SVM weights in terms of *in vivo* TF binding detection for JUND and CTCF in K562 cell line using AUC of ROC and PRC.

| Motif | Score | AUC Measure | method | |
| --- | --- | --- | --- | --- |
|  |  |  | Maximum | Average |
| JUND | PWM score | ROC | 0.7436920 | 0.6531090 |
|  |  | PRC | 0.8037595 | 0.6214914 |
|  | SVM weight | ROC | **0.7786460** | **0.7830780** |
|  |  | PRC | **0.8062867** | **0.7607107** |
| CTCF | PWM score | ROC | **0.9246795** | 0.3209550 |
|  |  | PRC | **0.9174528** | 0.3903729 |
|  | SVM weight | ROC | 0.7997380 | **0.8162730** |
|  |  | PRC | 0.7830818 | **0.7666536** |

**Supplementary Table S4.** Summary of the Top 100 SNVs with the Highest DeltaSVM Scores. The impact of all SNVs within motif region was examined using different TF-based deltaSVM score. The 100 SNVs with the highest deltaSVM score for each TF are summarized in each sheet. As can be seen, the motif deltaPWM score poorly captured the *in vivo* TF binding impact of these SNVs while the large deltaSVM score indicated more probable binding affinity impact.

(see separate excel file)

**Supplementary Table S5.** Percentage of discordant SNVs among Functional SNV Enrichment Determined for Each Disease. The impact of disease-associated SNVs located inside motif sites was examined using gkm-SVM-based method. The results for different diseases were summarized in this table. Only a small proportion of SNV were identified as significant among all putative TF bindings, which indicated the PWM-based annotation method is not accurate enough. Furthermore, the percentage of discordant SNVs is averaged among selected motifs for each disease, respectively. Most diseases have around 80% of discordant SNVs.

| TF | AD | | | Asthma | | | Breast Neoplasms | | | Cardiovascular Diseases | | | Child Development Disorders Pervasive | | | Colorectal Neoplasms | | | Crohn Diseases | | | Lung Neoplasms | | | Obesity | | | Psoriasis | | | Type II Diabetes | | | Overall |
| --- | --- | --- | --- | --- | --- | --- | --- | --- | --- | --- | --- | --- | --- | --- | --- | --- | --- | --- | --- | --- | --- | --- | --- | --- | --- | --- | --- | --- | --- | --- | --- | --- | --- | --- |
|  | Pos^1^ | All | DIS^2^ | Pos^1^ | All | DIS^2^ | Pos^1^ | All | DIS^2^ | Pos^1^ | All | DIS^2^ | Pos^1^ | All | DIS^2^ | Pos^1^ | All | DIS^2^ | Pos^1^ | All | DIS^2^ | Pos^1^ | All | DIS^2^ | Pos^1^ | All | DIS^2^ | Pos^1^ | All | DIS^2^ | Pos^1^ | All | DIS^2^ |  |
| BCL11A | 13 | 299 | 0.957 | 11 | 251 | 0.956 | 17 | 329 | 0.948 | 0 | 42 | 1.000 | 0 | 81 | 1.000 | 3 | 93 | 0.968 | 12 | 312 | 0.962 | 2 | 51 | 0.961 | 3 | 70 | 0.957 | 11 | 299 | 0.963 | 5 | 155 | 0.968 |  |
| CTCF | 6 | 10 | 0.400 | 7 | 11 | 0.364 | 6 | 15 | 0.600 | 1 | 1 | 0.000 | 0 | 3 | 1.000 | 2 | 6 | 0.667 | 3 | 10 | 0.700 | 2 | 4 | 0.500 | 2 | 2 | 0.000 | 5 | 13 | 0.615 | 6 | 8 | 0.250 |  |
| EGR1 | 37 | 127 | 0.709 | 39 | 100 | 0.610 | 30 | 111 | 0.730 | 7 | 19 | 0.632 | 12 | 37 | 0.676 | 18 | 34 | 0.471 | 49 | 114 | 0.570 | 8 | 19 | 0.579 | 18 | 40 | 0.550 | 43 | 120 | 0.642 | 19 | 59 | 0.678 |  |
| GABPA | 29 | 106 | 0.726 | 18 | 78 | 0.769 | 36 | 123 | 0.707 | 4 | 21 | 0.810 | 8 | 30 | 0.733 | 7 | 36 | 0.806 | 28 | 96 | 0.708 | 6 | 18 | 0.667 | 8 | 24 | 0.667 | 18 | 101 | 0.822 | 14 | 49 | 0.714 |  |
| JUND | 44 | 214 | 0.794 | 29 | 168 | 0.827 | 36 | 191 | 0.812 | 1 | 19 | 0.947 | 4 | 52 | 0.923 | 10 | 73 | 0.863 | 42 | 189 | 0.778 | 3 | 23 | 0.870 | 5 | 49 | 0.898 | 34 | 205 | 0.834 | 22 | 93 | 0.763 |  |
| JUN | 16 | 119 | 0.866 | 22 | 116 | 0.810 | 18 | 135 | 0.867 | 0 | 8 | 1.000 | 9 | 43 | 0.791 | 4 | 39 | 0.897 | 16 | 108 | 0.852 | 0 | 13 | 1.000 | 3 | 25 | 0.880 | 17 | 94 | 0.819 | 9 | 60 | 0.850 |  |
| MAX | 48 | 309 | 0.845 | 28 | 277 | 0.899 | 43 | 326 | 0.868 | 2 | 42 | 0.952 | 11 | 94 | 0.883 | 10 | 77 | 0.870 | 31 | 315 | 0.902 | 6 | 38 | 0.842 | 10 | 84 | 0.881 | 38 | 296 | 0.872 | 14 | 162 | 0.914 |  |
| NANOG | 5 | 299 | 0.983 | 12 | 251 | 0.952 | 16 | 329 | 0.951 | 2 | 42 | 0.952 | 2 | 81 | 0.975 | 4 | 93 | 0.957 | 12 | 312 | 0.962 | 4 | 51 | 0.922 | 1 | 70 | 0.986 | 5 | 299 | 0.983 | 7 | 155 | 0.955 |  |
| POU5F1 | 18 | 693 | 0.974 | 20 | 534 | 0.963 | 22 | 634 | 0.965 | 1 | 76 | 0.987 | 9 | 207 | 0.957 | 6 | 188 | 0.968 | 19 | 512 | 0.963 | 3 | 69 | 0.957 | 4 | 172 | 0.977 | 17 | 538 | 0.968 | 9 | 302 | 0.970 |  |
| RAD21 | 15 | 45 | 0.667 | 11 | 43 | 0.744 | 15 | 49 | 0.694 | 4 | 8 | 0.500 | 7 | 18 | 0.611 | 4 | 15 | 0.733 | 15 | 51 | 0.706 | 4 | 11 | 0.636 | 3 | 8 | 0.625 | 16 | 50 | 0.680 | 13 | 35 | 0.629 |  |
| RFX5 | 11 | 80 | 0.863 | 7 | 68 | 0.897 | 12 | 91 | 0.868 | 1 | 9 | 0.889 | 6 | 33 | 0.818 | 4 | 32 | 0.875 | 11 | 89 | 0.876 | 0 | 12 | 1.000 | 2 | 19 | 0.895 | 14 | 109 | 0.872 | 7 | 43 | 0.837 |  |
| SIX5 | 4 | 24 | 0.833 | 2 | 14 | 0.857 | 3 | 17 | 0.824 | 0 | 1 | 1.000 | 0 | 5 | 1.000 | 2 | 7 | 0.714 | 5 | 16 | 0.688 | 1 | 4 | 0.750 | 1 | 4 | 0.750 | 4 | 20 | 0.800 | 2 | 6 | 0.667 |  |
| SRF | 9 | 48 | 0.812 | 8 | 35 | 0.771 | 7 | 40 | 0.825 | 0 | 4 | 1.000 | 4 | 12 | 0.667 | 3 | 14 | 0.786 | 6 | 31 | 0.806 | 0 | 2 | 1.000 | 3 | 15 | 0.800 | 3 | 30 | 0.900 | 3 | 25 | 0.880 |  |
| STAT1 | 3 | 152 | 0.980 | 9 | 125 | 0.928 | 9 | 158 | 0.943 | 2 | 19 | 0.895 | 0 | 38 | 1.000 | 2 | 41 | 0.951 | 6 | 136 | 0.956 | 0 | 19 | 1.000 | 2 | 48 | 0.958 | 9 | 139 | 0.935 | 4 | 91 | 0.956 |  |
| TCF12 | 39 | 397 | 0.902 | 39 | 339 | 0.885 | 39 | 412 | 0.905 | 1 | 41 | 0.976 | 15 | 126 | 0.881 | 11 | 126 | 0.913 | 35 | 368 | 0.905 | 3 | 54 | 0.944 | 15 | 114 | 0.868 | 39 | 421 | 0.907 | 20 | 201 | 0.900 |  |
| USF1 | 57 | 130 | 0.562 | 26 | 73 | 0.644 | 58 | 127 | 0.543 | 11 | 21 | 0.476 | 19 | 34 | 0.441 | 13 | 55 | 0.764 | 52 | 110 | 0.527 | 9 | 24 | 0.625 | 14 | 31 | 0.548 | 62 | 118 | 0.475 | 25 | 58 | 0.569 |  |
| USF2 | 32 | 112 | 0.714 | 26 | 96 | 0.729 | 36 | 119 | 0.697 | 8 | 17 | 0.529 | 11 | 31 | 0.645 | 9 | 47 | 0.809 | 33 | 110 | 0.700 | 8 | 23 | 0.652 | 7 | 30 | 0.767 | 33 | 110 | 0.700 | 18 | 61 | 0.705 |  |
| YY1 | 24 | 107 | 0.776 | 17 | 89 | 0.809 | 21 | 106 | 0.802 | 5 | 18 | 0.722 | 7 | 27 | 0.741 | 3 | 33 | 0.909 | 27 | 95 | 0.716 | 0 | 7 | 1.000 | 7 | 22 | 0.682 | 28 | 107 | 0.738 | 15 | 60 | 0.750 |  |
| Average DIS^­­2^ |  |  | 0.798 |  |  | 0.801 |  |  | 0.808 |  |  | 0.793 |  |  | 0.819 |  |  | 0.829 |  |  | 0.793 |  |  | 0.828 |  |  | 0.761 |  |  | 0.807 |  |  | 0.775 | 0.801 |

**^1^ “Pos” indicates the disease-associated SNVs located inside motif sites identified by gkm-SVM-based method**

**^2^ DIS: Percentage of discordant SNVs.**
